# Design and method considerations for equity-centered implementation science and practice: A PEDALs approach

**DOI:** 10.1016/j.ghrp.2026.06.005

**Published:** 2026-06-22

**Authors:** Run (Sherry) Wang, Wenjun He, Lijing Yan, Rohit Ramaswamy, Judith N. Wasserheit, Mosoka P. Fallah, Ntuli Kapologwe, Malabika Sarker, Yiyuan Cai, Blandina Theophil Mmbaga, Dan Wu, Donna Spiegelman, Jia Bainga Kangbai, Easmon Otupiri, Mathuros Tipayamongkholgul, Dong (Roman) Xu

**Affiliations:** a Southern Medical University Institute for Global Health (SIGHT), Dermatology Hospital of Southern Medical University (SMU), Guangzhou, China; b Acacia Lab for Implementation Science, School of Health Management and Dermatology Hospital, Southern Medical University, Guangzhou, China; c Center for World Health Organization Studies and Department of Health Management, School of Health Management of Southern Medical University, Guangzhou, China; d Global Health Research Center, Duke Kunshan University, Kunshan, China; e James M Anderson Center for Health Systems Excellence, Cincinnati Children’s Hospital Medical Center, Cincinnati, Ohio, USA; f Medical School, University of Washington, Seattle, Washington, USA; g Africa Centres for Disease Control and Prevention, Science and Innovation Directorate, Addis Ababa, Ethiopia; h East, Central, and Southern Africa Health Community (ECSA-HC), Arusha, Tanzania; i Brown School of Public Health, Brown University, RI, USA; j Heidelberg Institute of Global Health, Heidelberg University, Germany; k Acacia Lab for Primary Healthcare, Department of epidemiology and health statistics, School of Public Health, Guizhou Medical University, Gui’an, China; l The key Laboratory of Environmental Pollution Monitoring and Disease Control, Ministry of Education, Guizhou Medical University, Gui’an, China; m Kilimanjaro Christian Medical Center, Moshi, Tanzania; n Department of Social Medicine and Health Education, School of Public Health, Nanjing Medical University, Nanjing, China; o Center for Methods in Implementation and Prevention Science, Department of Biostatistics, Yale School of Public Health, Yale University, New Haven, CT, USA; p Department of Biostatistics, Yale School of Public Health, Yale University, New Haven, CT, USA; q Department of Public Health, Njala University, Bo City, Sierra Leone; r Department of Population, Family and Reproductive Health, Kwame Nkrumah University of Science and Technology, Kumasi, Ghana; s ASEAN Institute for Health Development, Mahidol University, Nakhon Pathom, Thailand

## Abstract

The persistent "know-do gap"—the lag between the development of evidence-based practices (EBPs) and their routine use in health systems—remains a barrier to achieving global health equity. Implementation science offers a pathway to bridge this gap, but without an explicit equity focus, it risks reinforcing existing disparities. In this perspective, we propose an equity-centered adaptation of the PEDALs model (Problem, Evidence-based practice, Determinants, Action, Long-term use, and scale) to guide more just and context-sensitive implementation efforts in global health. We describe how each stage of PEDALs can be used to embed equity considerations in implementation research and practice. These include identifying root causes of inequity-linked health problems (P), using both scientific and practice-based evidence while adapting or de-implementing interventions (E), engaging marginalized voices when analyzing barriers and facilitators (D), co-designing contextually grounded implementation strategies (A), and measuring long-term implementation outcomes through an equity lens (L), including attention to scalability, iterative learning, and proper design and methods (s). We highlight methodological considerations—such as hybrid designs, embedded and workflow-based research, and rapid and participatory methods—that support timely, relevant, and equitable implementation. Our model builds on the concept of “radical incrementalism” to emphasize steady, equity-driven change that is responsive to diverse settings, especially in low- and middle-income countries. This modified PEDALs-based approach offers a practical structure for design and methodological considerations in advancing equity-centered implementation science.

## Background

The persistent "know-do gap", in which evidence-based practices (EBPs) take years to translate into routine care, severely impedes global health progress.^1^ Implementation science has emerged to bridge this gap by exploring the factors, strategies, and contexts that facilitate or hinder the implementation of EBP.^1^ However, implementation efforts are not neutral; without an explicit focus on equity, they risk favoring privileged populations and inadvertently widening existing disparities.^2^ We align with the World Health Organization’s vision of equity—not merely as a goal, but as the systematic removal of unfair, avoidable, or remediable health differences among socially or geographically defined groups.^3^ This requires more than a broad commitment; it demands a nuanced lens that addresses how ethnicity, age, gender, rurality, and disability intersect, as equity challenges often manifest uniquely at these overlapping boundaries.^4^

While earlier guidance was limited, the field has rapidly evolved with recent scoping reviews identifying a growing number of equity-focused theories, models, and frameworks (TMFs). Key foundational frameworks have been updated to center justice; for instance, the RE-AIM 2.0 framework now explicitly integrates equity across all dimensions,^5^ while the updated Consolidated Framework for Implementation Research (CFIR 2.0) has refined its constructs to better capture social determinants.^6^ Furthermore, vital literature on Indigenous-focused TMFs—such as the *He Pikinga Waiora* framework—emphasizes self-determination and the centering of Indigenous knowledge systems.^7^ Throughout this perspective, we employ an "equity lens," defined as a systematic evaluative process that interrogates how decisions, power structures, and resource allocations impact marginalized populations to eliminate structural disadvantages.

## The PEDALs model

**Fig. 1:**
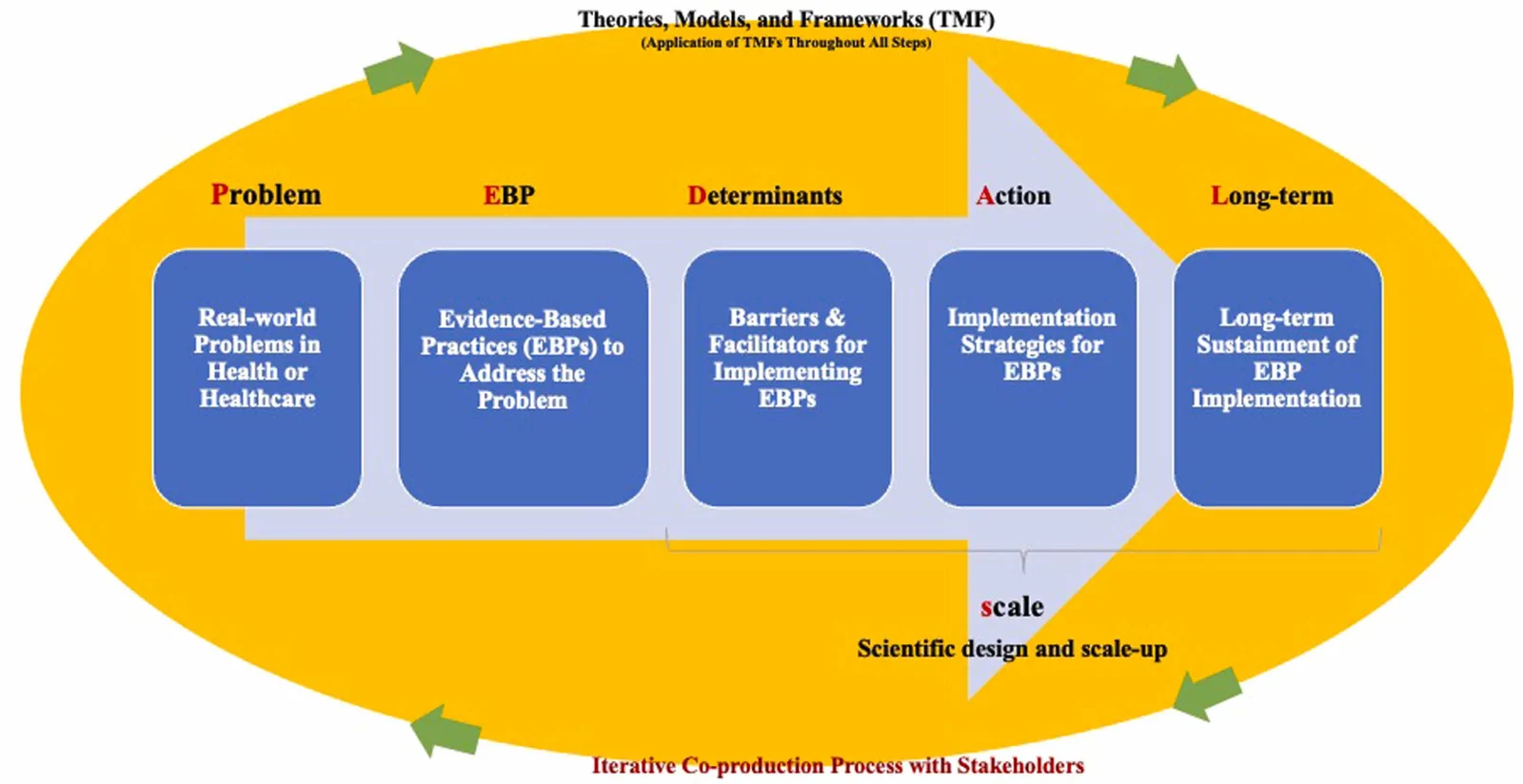
The PEDALs (Problem, Evidence-based/informed practice, Determinants, Action, Long-term use, and scientific design and scale-up) framework for equity-centered implementation science and practice in global health.

Based on his research and teaching experiences, Professor Dong Xu developed the initial PEDALs (Problem, Evidence-based practice, Determinant, Action, Long-term use, and scale) model, which was primarily conceived as a high-level pedagogical and practice-based scaffold rather than a model derived from formal theoretical deduction. Its origins in the classroom and field allow it to serve as an accessible framework for students and practitioners to navigate complex implementation cycles.^8^ While the original PEDALs was not designed as an equity-explicit model, the equity-centered adaptation we propose here reframes each of its components through an equity lens. In this adapted version, equity is not an optional add-on, but a core thread woven through every stage.^9^ This adaptation fills a critical gap in implementation science: a simple, step‑by‑step roadmap that embeds equity into every phase and makes existing TMFs more accessible to practitioners, especially in LMICs. The acronym symbolizes pedaling a bicycle—each stroke builds progress toward radical, sustainable change through "radical incrementalism".^10^ Each letter represents a sequential step in equity-centered practice (Fig. 1). The PEDALs model has since undergone continuous refinement based on insights from systematic reviews, user feedback, and expert consultation.

## P - Problem: equity-rooted identification

The problem in PEDALs is the health or healthcare challenges that must be addressed through implementation efforts. The problem that requires attention can be explicitly linked to equity, such as the stark rural-urban divide for maternal and neonatal mortality in Tanzania (a health problem) and the underuse of mental health services by urban slum residents in India despite their higher risks (a healthcare problem). However, implementation often targets problems not framed as equity challenges—like the overall low immunization coverage in Sub-Saharan Africa—when, in fact, these problems disproportionately affect underserved or vulnerable populations and thus require an equity lens in particular. To define the "problem" with an equity lens, researchers must first distinguish between the unique geopolitical contexts of implementation. In low- and middle-income countries (LMICs), health problems are often shaped by the legacies of colonial medicine and global economic dependencies that dictate resource distribution.^11^ In contrast, equity challenges in high-income countries (HICs) frequently center on dismantling structural racism and addressing the exclusion of minoritized groups from established national health systems.^12^ For Indigenous populations globally, health challenges are a matter of fundamental rights and sovereignty. This necessitates a "decolonizing" lens that moves beyond Western-centric methodologies to ensure research does not replicate colonial power imbalances.^12^

Regardless of whether equity is the central theme, the "problem" in PEDALs demands a focus on root causes, not just surface symptoms. Such root causes often extend beyond the health system and encompass broader social determinants, including poverty, geographic marginalization, ineffective governance, and ingrained cultural practices. For instance, childhood stunting in Iringa, Tanzania, seemingly a problem of food shortages, was rooted in dietary practices where nutrient-rich foods are avoided due to cultural beliefs or sold for income.^13^ This process relies heavily on community intelligence as a valid evidence base to define the problem itself, ensuring challenges are understood through the eyes of those most affected rather than solely through external epidemiological data. Furthermore, defining the "problem" requires a shift away from biomedical definitions of health as the mere absence of disease. For many Indigenous and minoritized populations, health is a holistic concept of wellbeing encompassing spirituality, mental health, and linkages to land and the environment.^14^ Implementation efforts must therefore acknowledge these population-specific models to ensure the identified "problem" aligns with the community’s own priorities and ontological understandings of wellness. Simple methods like the “5 whys” can help identify root problems by repeatedly asking "why." Specialized frameworks like *He Pikinga Waiora* can be instrumental at this stage, as they offer structured, Indigenous-focused approaches for community-led problem identification and the co-design of contextually grounded solutions.^7^

## E – evidence-based/informed practice: the “thing” to be implemented

Unlike traditional research, which often seeks novel interventions, implementation science prioritizes the application of existing EBPs to address identified problems above. This approach is more resource-efficient and ethically sound.^15^ Identifying the most suitable EBP for a given context is paramount. Below, we discuss three considerations that have significant equity implications.

First, what constitutes valid evidence for justifying an EBP, particularly in equity-focused global health contexts? While randomized controlled trials (RCTs) are highly valued in biomedical science, their generalizability across diverse contexts—a crucial consideration for equity-centered implementation—is limited.^16^ In many global health settings, rigorous local RCTs are often not feasible. Therefore, practice-based evidence—knowledge generated directly from real-world practices, settings, and communities—should also be considered.^17^ Moving further, we argue that “evidence” should explicitly encompass community and cultural intelligence. This includes the sophisticated, lived knowledge held by local populations regarding their social structures, spiritual health, and historical contexts.^18^ Rather than being relegated to mere contextualizing factors for RCT data, these forms of intelligence serve as primary evidence for determining what constitutes a best or suitable practice in a specific equity-driven setting.^18^ Consequently, although EBP remains an established term in implementation science, we advocate for the term evidence‑informed practice in global health settings, which more fully incorporates practical knowledge and contextual perspectives, while still maintaining a standard that excludes non‑scientific prejudices and superstitions.^19^ This inclusivity is a critical step toward decolonizing knowledge because it acknowledges local and Indigenous wisdom alongside traditional scientific evidence, while countering the historical marginalization of non-Western knowledge systems.

This leads to the second critical aspect: adapting and optimizing EBPs for local contexts. Adaptation is the deliberate modification of an intervention to enhance its fit in a new context.^20^ Adaptation is critical in improving health equity. For instance, many EBPs originated from the Global North and are often too costly; therefore, a failure to adapt may perpetuate an overreliance on development aid and contribute to global health colonization. To systematically document such changes and share lessons learned, reporting frameworks like FRAME (for interventions)^21^ and the emerging FRAME-IS (for implementation strategies)^22^ provide structured approaches to capture what was adapted, why, and by whom, helping the field distinguish generalizable principles from context-specific adaptations. Optimization, as described by Professor Linda Collins in her seminal work on Multiphase Optimization Strategy,^23^ can be considered a form of adaptation aimed at modifying a multi-component intervention to achieve an optimal configuration, one that leads to the best outcome under contextual constraints rather than an unconditional best outcome. Depending on available resources, optimization can range from full optimization trials—often using efficient factorial designs—to more rapid approaches, such as survey-based discrete choice experiments or simply stakeholder consultation to adjust intervention components .^23^ Within this optimization framework, identifying core elements of multi-component interventions is essential for achieving equitable outcomes, while allowing non-core components to be adapted locally without compromising effectiveness or fairness.

The third, often overlooked, issue is the de-implementation of ineffective, low-value, or potentially harmful practices.^24^ This is directly linked to equity: marginalized populations may experience a dual burden of limited access to effective services and disproportionate exposure to low-value, costly, or potentially harmful care. For example, inappropriate injectable therapies and unnecessary antibiotic or corticosteroid use in under-resourced primary care settings illustrate practices that are not supported by evidence and may increase out-of-pocket costs, adverse effects, and antimicrobial resistance.^25^ In China, the colloquial term “three ingredients in one soup” has been used to describe the combined use of antibiotics, corticosteroids or hormones, and vitamins in glucose infusions for undifferentiated minor illness; this example is included to illustrate why equity-centered implementation must include de-implementation, not only the adoption of new EBPs.^26^ De-implementation strategies such as audit and feedback, clinician communication training, patient education, antibiotic stewardship, and locally feasible alternatives are therefore as important as implementation strategies for EBPs. Furthermore, in regions where implementation science is emerging, we should carefully label interventions as "evidence-based”, as it risks inappropriately integrating non-EBPs into routine care and thus inadvertently reinforces low-value care and further widens health inequities.

## D - Determinants to implementation: barriers and facilitators

Once an EBP, or candidate EBP, has been identified and optimized, the subsequent crucial step involves systematically investigating the determinants—both barriers and facilitators—to its successful implementation. Implementation science is well-equipped with TMFs and empirical studies in this area. Among these, the Consolidated Framework for Implementation Research (CFIR) ^6^ is widely used. CFIR consolidates existing constructs related to implementation determinants into five broad domains: innovation, outer setting, inner setting, individuals, and implementation process. In investigating the determinants of implementation, it is critical to engage diverse stakeholders to identify voices from marginalized and underrepresented groups. This includes identifying who benefits from an EBP, who does not, and why. The process should ideally be guided by TMFs, such as the CFIR adapted to include equity considerations or specifically equity-oriented TMFs, like the Health Equity Implementation Framework (HEIF).^27^ The PEDALs also provide the flexibility to integrate Indigenous-led frameworks or process-oriented tools, which ensure that the investigation of determinants moves beyond surface-level barriers to address historical and structural power imbalances. Additionally, researchers can utilize emerging TMF selection tools that incorporate equity-driven algorithms or reflective questions to ensure the chosen framework is fit-for-purpose in marginalized contexts.

Such approaches enable explicit assessment of structural (e.g., income, education, housing—many of which are social determinants of health), cultural, and interpersonal factors that impact equity. Furthermore, methods should also be applied to ensure that these TMFs are used in a way that stimulates genuine reflexivity and power-sharing. Practitioners should employ critical theoretical approaches to interrogate how historical injustices and ongoing colonial structures act as determinants of determinants, influencing who holds the power to define problems and select solutions.^11^, ^12^ Otherwise, researchers may unintentionally perpetuate inequities through their biases and decision-making processes (further elaborated in the co-production methods section).

A frequently observed but increasingly contested tendency in implementation research, particularly in low- and middle-income country settings, is the framing of local beliefs, practices, or traditions as “cultural barriers” to evidence-based practice uptake.^28^ We argue for a critical re-examination of this construct. So-called cultural barriers are frequently symptoms of deeper structural issues—such as systemic neglect, a lack of trust in colonial-inherited institutions, or the fundamental misalignment of a Western-developed EBP with local realities.^28^ Rather than viewing culture as an obstacle to be overcome, implementation science must prioritize cultural safety and the reflexivity of the implementers. This shift ensures that the analysis of determinants interrogates whether the intervention itself is contextually inappropriate or extractive, rather than blaming the community's culture for implementation failures. By reframing determinants through a lens of power and history, PEDALs ensures that implementation strategies (A) are built on a foundation of structural justice rather than cultural deficit models.

## A - Action to address the determinants: implementation strategies

The purpose of identifying the determinants of implementation is to inform the next steps in developing and applying implementation strategies that address those determinants. It is crucial to distinguish between the EBP itself and the strategy employed for its implementation. This distinction is context-dependent: for instance, a brief verbal intervention might be a strategy to increase flu vaccination (the EBP), while economic incentives could be a strategy to promote clinician uptake of that same verbal intervention (now considered the EBP, if proven effective in previous implementation research).

Two complementary approaches typically guide the selection of strategy. A practice-oriented approach prioritizes context-specific strategies to overcome identified implementation determinants, often facilitated by tools such as the CFIR-ERIC matching tool.^29^ These strategies may not be innovative but have some evidence supporting their general effectiveness. Conversely, a research-oriented approach focuses on testing innovative strategies with unknown effectiveness, such as the “pay-it-forward” model (as a new strategy) for promoting gonorrhoea and chlamydia testing (an EBP) among men who have sex with men.^30^ The former aims for immediate application of effective strategies, while the latter seeks generalizable knowledge about new and novel strategies. In either case, equity-conscious co-design with diverse stakeholders is vital in developing these strategies.

For implementation studies, the innovativeness often lies not in the EBP itself, but in the strategies used to implement it. Innovative implementation strategies may draw on diverse sources. We can learn directly from end-users and communities, who frequently possess effective, context-specific solutions to local challenges, such as “test and trust exchange” for HIV testing.^31^ Participatory methods, such as crowdsourcing, help elicit such insights. Furthermore, successful innovations from commercial or other sectors can be adapted; for instance, the “pay-it-forward” model, originally a business concept for coffee shops, has been successfully applied to promote gonorrhoea and chlamydia testing.^30^, ^32^

Equity-oriented implementation inherently demands the development of context-specific strategies, which presents a challenge for research aiming to produce generalizable knowledge. This is because hypothesis testing for intervention effectiveness often requires the intervention to be fixed and uniform across diverse groups and settings. One solution to navigate this challenge is to define interventions by their function (the overarching aim, e.g., providing patient education), content (the specific components or information, e.g., diabetes self-care), and form (the method or setting of delivery, e.g., online versus in-person delivery).^33^ With this approach, fidelity can be maintained and assessed at the functional level, while allowing for contextual adaptation in content and forms.^34^ For example, a multi-country implementation on community health worker (CHW) programs can uphold the function of improving care access for underserved communities while tailoring the content of services delivered (such as maternal health support or chronic disease management) and the delivery forms (like in-person home visits, community workshops, or telehealth). This approach is essential for addressing health equity, as it better meets the diverse needs of different populations while maintaining the overarching goal.

## L - Long-term use of EBP in practice routines

The goal of implementation is the long-term use of EBPs in routine care, which requires the systematic measurement of implementation outcomes, particularly through an equity lens. In contexts where holistic health models prevail, these outcomes should move beyond clinical indicators to include metrics that reflect spiritual or communal wellbeing and environmental harmony.^27^ Measuring the success of an intervention through an equity lens necessitates evaluating whether the EBP supports the broader flourishing of the community as defined by their own cultural standards.^27^ While established outcome frameworks, such as the original RE-AIM ^5^ and Proctor’s outcomes,^35^ provide a strong foundation but lack explicit attention to equity. However, the updated RE‑AIM 2.0 framework directly addresses this limitation by explicitly centering equity—assessing not just reach but equitable reach and evaluating the distribution of effectiveness across diverse populations.^5^ Despite this limitation, we propose using those existing outcome frameworks rather than relying on equity-specific frameworks. This is because we consider any outcome to have two dimensions: the absolute attainment (the mean value of the entire population) and distribution across groups. The latter naturally provides a comprehensive assessment of equity (e.g., whether Proctor dimensions—acceptability, appropriateness, feasibility, and fidelity—vary across advantaged and disadvantaged groups).

Although we do not advocate for creating new equity-specific implementation outcome frameworks, existing ones also present limitations. For instance, while Proctor emphasizes *sustainability* and RE-AIM emphasizes *maintenance*, these two concepts are distinct yet equally important: sustainability refers to the likelihood of continued EBP use into the future, whereas maintenance reflects the actual sustained use at a particular time point. Crucially, both concepts are deeply connected to equity and the broader goal of sustaining impact over time, especially in under-resourced settings. Furthermore, both frameworks overlook scalability—the likelihood of expanding an EBP to new contexts or populations—and scale-up—the actual expansion of the program. Scalability and scale-up are critical for extending the benefits of effective interventions to a broader range of disadvantaged groups and thus warrant considerably more empirical investigation in global health.

Another key consideration is assessing acceptability, appropriateness, and feasibility. These three constructs are essential mediators that are conceptually and empirically linked to both sustainability and scalability. However, these constructs are not explicitly integrated into the RE-AIM framework and often overlap conceptually, making it challenging to disentangle them in practice (e.g., something deemed acceptable is also likely to be considered appropriate). To simplify this matter, we propose combining those three concepts into a single construct (implementability or feasibility), which can be assessed using validated scales, such as the Theoretical Framework of Acceptability (TFA).^36^ However, in cross-cultural contexts, we must caution against the risks of such reductionism. For many populations, acceptability is not a static numerical score but a dynamic, relational construct. Therefore, while validated scales provide a useful baseline, they must be complemented by interpretive, qualitative methods that capture deep-seated cultural, spiritual, and social meanings. Finally, research must operationalize these outcomes with specific indicators, such as quality checklists, and appropriate measurement tools like standardized patients. Crucially, the governance of this data must respect community-determined data sovereignty. Equitable implementation requires that communities have the power to own, control, and access their own data, ensuring that the evaluation process does not inadvertently perpetuate extractive research practices.

## s – scientific design and scale up

The "s" in PEDALs has triple meanings: first, it denotes the plural form of PEDAL, the potential for multiple, iterative implementation cycles towards radical change; second, it explicitly emphasizes scaling up as a distinct endpoint that goes beyond mere sustainment in the initial setting or population; and third, it pertains to scaling (ie., evaluating) the effectiveness of the implementation using appropriate design and methods. We have discussed the first two points in prior sections. Below, we focus on several key equity considerations related to the third point (ie., the overall design and methods within PEDALs).

First, central to equity-centered implementation is the integration of participatory research and co-production across all stages of the PEDALs model. However, to avoid tokenistic engagement, this must evolve into genuine community leadership supported by long-term relationships and investment in local capacity and capability building. Researchers have a responsibility to foster their own critical consciousness and cultural safety, acknowledging and respecting local knowledge holders as equals in the research process. Engaging diverse stakeholders, particularly underrepresented groups, is crucial: it ensures that identified problems (P) genuinely reflect community needs, that selected EBPs (E) are relevant and appropriate, that implementation determinants (D) are accurately identified and relevant to stakeholders, that action strategies (A) foster ownership and effectiveness, and that long-term implementation outcomes (L) are meaningful and achievable. To enhance inclusivity, methods accommodating varying educational levels—such as storytelling, role-playing, simulations, and creative arts-based approaches (e.g., drawing, photography, theatre)—should be considered. This process often requires consensus-building, in which methods such as the Delphi method, crowdsourcing, Nominal Group Technique, Designathons, and iterative prototyping can be useful for fostering a shared understanding and prioritization. However, it is crucial to recognize that co-design is not always the most appropriate or feasible approach in every context. Implementation research should consider a spectrum of participation, where the level of community engagement—ranging from informing and consulting to deep co-production—is determined by a combination of project resources, timelines, and the community’s own stated preference for involvement. Furthermore, mixed methods are often valuable, particularly for understanding the complex social and structural contexts that influence implementation. For instance, focus groups can qualitatively generate a comprehensive list of barriers (providing deeper insight from a limited group) that are then quantitatively prioritized (e.g., using best-worst scaling survey) with a larger, more representative sample.

Second, we advocate for the widespread adoption of hybrid designs in effectiveness trials to reduce delays in delivering effective health interventions to broader (often disadvantaged) populations.^37^ Traditionally, research progresses sequentially from efficacy to effectiveness and then to implementation studies—a process that is often protracted. Hybrid designs (Types 1, 2, and 3) overcome this by integrating effectiveness and implementation research, simultaneously investigating the candidate EBP and its implementation strategy, with a progressive focus on implementation outcomes. Hybrid 2 trials are particularly relevant for advancing equity. Many EBPs developed and evaluated in high-income settings require adaptation and reevaluation in the Global South. These EBPs, however, have a higher likelihood of proving effective compared to entirely new interventions that have not been evaluated. Therefore, conducting effectiveness and implementation evaluations simultaneously, rather than sequentially, saves both time and resources to reach these interventions to the Global South. In such scenarios, the dual RCT design, which concurrently evaluates both the intervention and its implementation strategies, can be beneficial, although it is rarely used. For example, a dual three-arm RCT ^38^ could compare (1) electronic pillbox for tuberculosis management (EBP alone), (2) no EBP, and (3) EBP with economic incentives (an implementation strategy), thereby assessing both EBP effectiveness (1 vs 2) and strategy effectiveness (1 vs 3).

Third, we echo the World Health Organization’s call for embedded research - embedding of research in real-world policy practice and program implementations.^39^ Embedding research naturally connects researchers with policymakers and implementers, thereby improving the uptake of research findings in routine practice .^40^ Additionally, we propose "workflow-based research" as a specific form of embedded research that transforms foundation or government-supported programs into large-scale implementation research. In this approach, interventions - whether EBPs or strategies - are already funded as part of the program delivery. Researchers simply need to carefully plan for participant allocation (random or non-random) and pre-specify outcomes and their measurement methods. Crucially, data collection should align seamlessly with existing workflows, meaning data is automatically generated through task completion rather than as a separate, burdensome process. For instance, our team is collaborating with the Guangzhou Center for Disease Control and Prevention to randomly assign residents to one of eight groups. These groups feature various combinations of sodium reduction strategies in a factorial design. All outcomes will be derived from the routine program reporting system. Workflow-based research requires only minimal dedicated funding, as the deployment of interventions and data collection primarily follows the program workflow. This is particularly important given the declining support for research in global health.

Lastly, we support the use of rapid methods over conventional approaches. Implementation science has developed or adapted conventional methods into rapid methods that require fewer resources, less training for data collectors and analysts, and enable rapid cycles of implementation in real-world settings. Empirical evidence suggests that rapid qualitative methods can yield results comparable to traditional methods,^41^ For example, a deductive rapid CFIR approach required fewer analyst hours and eliminated transcription costs while effectively meeting evaluation objectives and establishing rigor.^42^ Rapid methods also extend to quantitative approaches; for instance, Best-Worst Scaling imposes less cognitive burden than discrete choice experiments (DCEs), while DCEs can serve as proxies for trials when trials are infeasible.

Despite the abundance of implementation TMFs, PEDALs offer a unique practical scaffold. It provides granular, iterative guidance for equity-centered practice: PEDALs act as a meta-framework that translates equity concepts into actionable steps across the implementation lifecycle. Compared to QUERI's general roadmap,^43^ PEDALs explicitly embed equity into each sequential step (P, E, D, A, L, s) and operationalizes "radical incrementalism." Designed as a pedagogical and practice-based tool, it is particularly accessible for LMIC practitioners and students. Although still emerging, PEDALs has been applied in chronic wound care, perinatal care, and postoperative rehabilitation in China; future empirical comparisons with other frameworks are a valuable direction.

## Conclusions

The persistent "know-do gap", exacerbated by systemic inequities, necessitates an equity-centered approach to implementation science. Our proposed PEDALs model provides a structure for "radical incrementalism" towards equitable change. We advocate for the explicit integration of equity across all stages of PEDALs, using proper design and methods: P (equity-rooted Problem identification), E (Evidence-based/informed practice identification and de-implementation), D (Determinants investigation with explicit focus on equity), A (Action strategies co-designed for equity), L (Long-term EBP use (sustainment) measured by disaggregated equity analysis), and S (scaling up and evaluation to ensure higher equity). To illustrate how this model can be applied in practice, Table 1 presents an application of the PEDALs framework to the Better Birth Program in Uttar Pradesh, India, with an explicit equity lens.^44^ We are living in a tumultuous time for global health. Promoting health equity has never been more important, and as we continue to "pedal" forward with equity-centered implementation science, we can still forge a path toward lasting change and reducing inequity.

**Table 1:** Applying the PEDALs Model to The Better Birth Program with an Equity Lens.

**PEDALs**	**Core Concept**	**Example from The Better Birth Program**	**How Equity Was Incorporated**	**How Equity Could be Incorporated**
P - Problem	Health or healthcare challenge to be addressed.	High maternal and neonatal morbidity and mortality in Uttar Pradesh, India, despite increased facility-based childbirth. This points to a "know-do" gap in adherence to essential birth practices.	The problem itself highlights a disparity (high mortality/morbidity in a specific, often underserved, region). Equity was implicitly addressed by targeting a region with significant health challenges.	To explicitly enhance equity: ✔ Deeper analysis of which subgroups within Uttar Pradesh experienced the highest rates (e.g., rural vs. urban, specific castes, lowest income quintiles). ✔ Community-based participatory research to understand how different marginalized groups perceived the problem and its root causes (e.g., access barriers, cultural practices influencing early discharge).
E - Evidence-based/informed Practice	The "thing" to be implemented.	The WHO Safe Childbirth Checklist (SCC): A collection of 28 evidence-based essential birth practices known to improve quality and safety during childbirth. It was adapted to fit India's national guidelines.	The SCC is an evidence-based tool globally. Its adaptation to Indian national guidelines is a step towards contextual relevance, which supports equity. The "Read-Do" and "Do-Confirm" methods allowed flexibility in use by birth attendants.	To explicitly enhance equity: ✔ Systematic assessment of the SCC's cultural appropriateness and comprehensibility for diverse birth attendant literacy levels and local languages beyond just national guidelines. ✔ Consideration of "de-implementation" if any local practices related to SCC were found to be low-value or harmful for specific marginalized groups. ✔ Inclusion of qualitative data from birth attendants on their experiences with adapting the SCC to challenging, resource-constrained environments to ensure feasibility for all.
D - Determinants to implementation	Barriers and facilitators to successful implementation.	The Better Birth Program used a modified Opportunity-Ability-Motivation-Supplies (OAMS) framework to classify implementation determinants: ➢ Opportunity: Staffing levels, time, facility size. ➢ Ability: Provider knowledge, skills, communication. ➢ Motivation: Provider willingness to change behavior. ➢ Supplies: Availability of necessary medications, equipment. (e.g., misunderstanding about BCG vaccine vial usage).	The OAMS framework, by explicitly including "Supplies" and focusing on practical barriers, inherently addresses equity by identifying resource constraints common in underserved settings. The qualitative data collected captures lived experiences.	To explicitly enhance equity: ✔ Systematic identification of determinants specific to marginalized birth attendants (e.g., lower training access, heavier workload, cultural expectations). ✔ Use of an equity-oriented framework (like HEIF) in addition to OAMS to explicitly map how structural inequities contribute to these determinants (e.g., policies leading to understaffing in rural facilities). ✔ Ensuring that all levels of staff, including those from marginalized groups, feel empowered to identify and voice determinants.
A - Action to address the determinants	Implementation strategies to overcome identified determinants.	Coaching-Based Implementation strategy: ➢ Multilevel: Coaches for birth attendants; coach team leaders for facility/district leaders. ➢ Collaborative: Peer-to-peer, non-judgmental approach; invited active participation. ➢ Provider-centered: Focused on empowering providers to identify and solve their own problems using local resources (e.g., birth attendants and coaches agreeing to adapt discharge practices for early leaving mothers). ➢ Tasks: Increase motivation, observe/record/feedback (using Pulse app heat maps), and problem-solve.	The coaching strategy, with its emphasis on collaboration, provider-centeredness, and multilevel support, inherently fosters equity by building local capacity and tailoring solutions. Recruiting coaches from the "same hub" and using female nurses as coaches respects cultural norms and local contexts.	To explicitly enhance equity: ✔ Explicit co-design of coaching strategies with birth attendants from diverse backgrounds, ensuring methods are culturally sensitive and accessible (e.g., visual aids, storytelling). ✔ Strategies to ensure that solutions developed are feasible and sustainable for the most resource-constrained facilities or those with the highest staff turnover. ✔ Training for coaches on implicit bias and equitable communication to ensure all birth attendants receive the same quality of support regardless of background.
L - Long-term use of EBP in practice routines	Sustained use of the EBP and measurement of implementation outcomes.	Sustainability Plan: Coaching for empowerment and the Childbirth Quality Coordinator (CQC) role. CQCs were facility-based champions chosen for motivation and respect, not just title, and received coaching skills training. Measurement: Pulse (a mobile-friendly management information system) provided near-real-time data on SCC use, adherence to practices, and supply availability, displayed as heat maps.	The focus on empowerment and local champions is a strong equity principle, building sustainable capacity from within the community rather than relying on external support. The data feedback system (Pulse) allows for performance monitoring. Pulse also mirrors the concept of workflow-based research that minimized dedicated data collection efforts.	To explicitly enhance equity: ✔ Disaggregated analysis of outcomes: Instead of just overall adherence, examine SCC use and practice adherence across different birth attendant demographics (e.g., experience level, shift times, facility type) and patient outcomes (e.g., for different socio-economic patient groups). ✔ Tracking equity-specific outcomes: e.g., did the program reduce disparities in practice adherence between high- and low-volume facilities? ✔ Regular, participatory feedback loops from CQCs and birth attendants to ensure the sustainment strategies are truly working for the most vulnerable staff and patients.
s – scientific design and scale-up	Multiple cycles, explicit emphasis on scaling, and evaluating implementation effectiveness.	Scale-up: Program scaled to 60 facilities across 24 districts in Uttar Pradesh (approx. 60 million population). Attributed partially to the robust cloud-based data collection (Pulse). Iteration/Learning: Lessons learned about coaching principles (multilevel, collaborative, provider-centered) and need for adaptive coaching. Evaluation: Matched-pair, cluster-randomized controlled trial to test effectiveness.	Scaling the program to a large, diverse state like Uttar Pradesh inherently addresses equity by broadening access to improved care. The rigorous trial design provides robust evidence for effectiveness.	To explicitly enhance equity: ✔ Scale-up: Did the scale-up reach and benefit all intended populations equally? Were there unforeseen negative consequences for any group? ✔ iterative adaptation for equity: Use feedback from scale-up to further refine strategies to address challenges specific to previously unreached or more marginalized populations (e.g., adjusting coaching schedules for night shifts, addressing safety concerns for female coaches in remote areas) ✔ Evaluation/Methods: Using a dual RCT design would have strengthened the evaluation of the effectiveness of both SCC and its implementation strategies; Using embedded research will be highly useful in the scale-up stage within real-world settings. Additionally, many rapid qualitative and quantitative methods can be considered to facilitate quick and more inclusive stakeholder feedback.

## CRediT authorship contribution statement

**Run (Sherry) Wang:** Writing – review & editing, Writing – original draft, Conceptualization. **Yiyuan Cai:** Writing – review & editing. **Malabika Sarker:** Writing – review & editing. **Ntuli Kapologwe:** Writing – review & editing. **Mosoka P Fallah:** Writing – review & editing. **Dong (Roman) Xu:** Writing – review & editing, Conceptualization. **Judith N Wasserheit:** Writing – review & editing. **Mathuros Tipayamongkholgul:** Writing – review & editing. **Rohit Ramaswamy:** Writing – review & editing. **Lijing Yan:** Writing – review & editing. **Wenjun He:** Writing – review & editing, Visualization, Validation, Supervision, Methodology, Conceptualization. **Easmon Otupiri:** Writing – review & editing. **Jia Bainga Kangbai:** Writing – review & editing. **Donna Spiegelman:** Writing – review & editing. **Dan Wu:** Writing – review & editing. **Blandina Theophil Mmbaga:** Writing – review & editing.

## Ethics approval and consent to participate

Not applicable. This study is a conceptual and methodological analysis and did not involve human participants, human data, or biological materials.

## Consent for publication

Not applicable.

## Funding

This work was supported by the 10.13039/100009131Swiss Agency for Development and Cooperation (SDC) under grant number 81067392. The funder had no role in the study design, conceptual development, interpretation, or writing of this manuscript.

## Declaration of Generative AI and AI-assisted technologies in the writing process

During the preparation and revision of this work, the authors used DeepSeek-V4-Pro to assist with readability improvement. After using this tool, the authors reviewed and edited the content as needed and take full responsibility for the content of the published article.

## Declaration of Competing Interest

All other authors declare that they have no competing interests.

## Data Availability

No data was used for the research described in the article.
